# Generation of Case-Based Materials Using Large Language Models in Health Professions Education: Scoping Review

**DOI:** 10.2196/94795

**Published:** 2026-11-03

**Authors:** Zineb Zouakia, Alan Szymczak, Salomé Tordjman, Emmanuel Logak, Thomas Meyre, Matthieu Resche-Rigon, Alain Cariou, Anita Burgun, Rosy Tsopra

**Affiliations:** 1 Clinical Bioinformatics Laboratory, Imagine Institute, Université Paris Cité, INSERM UMR1163, 24 Bd du Montparnasse, 75015, Paris, F-75006, France, 33 668169844; 2 Department of Medical Informatics, Hôpital Européen Georges Pompidou, Hôpital Necker Enfants Malades, AP-HP, Paris, France; 3 Faculté de Santé, DigiHealth UPCité Team, Université Paris Cité, Paris, F-75006, France; 4 Biostatistics and Medical Information Department, Saint-Louis Hospital, Assistance Publique Hôpitaux de Paris, Paris, France; 5 Medical Intensive Care Unit, Cochin Hospital, Assistance Publique-Hôpitaux de Paris, Paris, France; 6 UPPERS, Université Paris Cité, US 007, Paris, F-75006, France

**Keywords:** large language models, generative AI, GenAI, health profession education, medical education, case-based learning, digital health, medical informatics

## Abstract

**Background:**

Large language models (LLMs) are increasingly used to generate case-based materials for health professions education (HPE). Previous reviews have examined broad applications in HPE or focused on clinical cases, but none have comprehensively synthesized the generation and evaluation of diverse case-based materials.

**Objective:**

This study aims to synthesize current evidence on LLM-generated case-based materials in HPE, focusing on generation approaches, evaluation methods, key findings, and reported challenges.

**Methods:**

This scoping review followed the Joanna Briggs Institute methodology and PRISMA-ScR (Preferred Reporting Items for Systematic Reviews and Meta-Analyses extension for Scoping Reviews) reporting guidelines. Seven databases (PubMed, Embase, Scopus, Web of Science, CINAHL, ERIC, and IEEE Xplore) were searched on May 10, 2026, for studies published from January 1, 2017. Eligible studies were peer-reviewed original research articles, published in English, reporting the use of generative LLMs to create text-based case-based materials for HPE. Data were synthesized using descriptive statistics and narrative synthesis.

**Results:**

Seventy-two studies were included. Most were published between 2024 and 2026 (67/72, 93%) and focused on medical education (59/72, 82%), with undergraduate students as the most frequently studied population. Case-based multiple-choice questions (MCQs; 28/72, 39%) and clinical cases or vignettes (22/72, 30%) were the most frequently generated materials. Generative Pretrained Transformer-4 (GPT-4) family models were the most commonly used (40/72, 55%), and generation predominantly relied on relatively simple prompting approaches, whereas more advanced strategies, such as few-shot prompting, prompt chaining, or use of external knowledge sources, were rarely adopted. Evaluation primarily relied on expert review, typically involving small panels, while student assessment (25/72, 35%) and psychometric analyses were less common. Overall, LLM-generated materials were frequently judged to be clinically plausible, structurally clear, and reasonably aligned with educational objectives, particularly after refinement. However, consistent limitations included factual inaccuracies, construction flaws, superficial clinical realism, pedagogical misalignment, suboptimal psychometric performance, and bias. No LLM consistently outperformed others, although methodological heterogeneity limits cross-study comparisons.

**Conclusions:**

This review provides a comprehensive synthesis of the literature on LLM-generated case-based materials in HPE. Unlike previous reviews examining broad educational applications or focusing on clinical cases, it integrates evidence across a broad range of case-based materials while synthesizing generation methods, evaluation approaches, reported findings, and challenges. The evidence suggests that LLMs can support the development of case-based materials but still require expert oversight because of recurring concerns regarding clinical accuracy, educational quality, psychometric performance, clinical realism, and bias. By identifying methodological and evidence gaps, this review provides educators, curriculum developers, and researchers with a structured overview of this rapidly evolving field and may inform the development of more robust generation workflows, more standardized evaluation approaches, and the responsible use of LLM-generated case-based materials in HPE.

## Introduction

### Rationale

The emergence of large language models (LLMs) is substantially transforming health education by introducing novel teaching methods and educational practices. Their potential is considerable and extends across multiple levels, including curriculum design, resource generation, personalized learning support, assessment support, and assistance with academic writing and bibliographic management [1-7].

In health professions education (HPE), one key challenge relates to the development of case-based educational materials, which are widely used to promote clinical reasoning, knowledge integration, and decision-making in authentic contexts [8]. For the purpose of this review, case-based materials were defined as text-based educational resources that present realistic clinical situations requiring learners to apply knowledge, interpret information, and make decisions [9]. These materials include clinical vignettes or cases [10], which summarize patient situations to support reasoning and discussion; case-based questions [11], which combine clinical cases with assessment items, such as multiple-choice questions (MCQs), and are commonly used in high-stakes examinations; and simulation scenarios [12], which consist of structured scripts designed for use in simulated environments. Evidence indicates that case-based approaches lead to greater improvements in learner knowledge and performance compared with traditional lectures [13-16]. However, the development of high-quality case-based materials is resource-intensive: cases must be clinically coherent, aligned with learning outcomes, appropriately challenging, and frequently refined through expert review and psychometric testing [17-19]. These requirements can limit the size, diversity, and timely updating of case libraries and assessment banks, particularly where faculty time or specialty expertise is limited.

LLMs offer novel opportunities for the generation of case-based educational materials. Recent reviews have examined the broader use of LLMs in HPE [1-3]. Lin et al [1] synthesized applications across medical education, including educational resources, teaching methods, and assessment, and highlighted both opportunities and implementation challenges. Basil et al [3] mapped the broad integration of generative AI (GenAI) across HPE, identifying applications in curriculum design, content creation and delivery, personalized learning, assessment, automated grading, and feedback. Similarly, Astbury et al [2] reviewed the use of GenAI for teaching and assessment across health professions and emphasized its growing role in learning resource development and educational assessment. These reviews demonstrate the expanding role of LLMs in HPE but address case-based generation only as 1 component of a broader educational landscape. Notably, Basil et al [3] identified content creation as the predominant area of GenAI integration in HPE, highlighting the growing importance of this application. Given the central role of case-based materials in HPE, a more focused and detailed synthesis of their generation using LLMs is therefore warranted.

A more focused scoping review by Kaya et al [20] examined LLM-generated clinical cases and vignettes. However, its scope was limited to cases and vignettes intended to function as standalone cases and explicitly excluded case-based MCQs and other formats combining clinical scenarios with assessment activities. In addition, although prompting and evaluation approaches were reported, their methodological characterization remained relatively broad, focusing mainly on prompt reporting and single versus iterative prompting, while evaluation findings were synthesized primarily in terms of overall case quality and bias. A more comprehensive and granular synthesis encompassing a broader spectrum of case-based educational materials and providing greater detail on their generation and evaluation processes therefore remains needed. The current review addresses this gap by encompassing clinical cases and vignettes, case-based questions, and simulation scenarios, and by providing a detailed characterization of the models and reference materials used, prompting and refinement approaches, evaluation methods and criteria, and reported findings and challenges across multiple quality domains.

### Objectives

The objective of this scoping review was to synthesize the current evidence on the use of LLMs to generate case-based materials in HPE. Specifically, the review sought to examine how these models were used to generate case-based educational materials, how the resulting materials were evaluated, and what findings and challenges were identified. To achieve this objective, the review addressed the following questions: (1) How are LLMs being used to generate case-based materials in HPE? (2) How are these LLM-generated case-based materials evaluated? (3) What key findings and challenges are reported in the existing literature?

## Methods

### Overview

This review was guided by the Joanna Briggs Institute methodology for scoping reviews and reported following the PRISMA-ScR (Preferred Reporting Items for Systematic Reviews and Meta-Analyses extension for Scoping Reviews) guidelines [21]. The completed PRISMA-ScR checklist is available in Checklist 1.

### Eligibility Criteria

#### Types of Participants

Studies were eligible if participants (ie, learners, educators, or programs involved) were within HPE.

#### Concept

The concept of interest was the use of generative LLMs to create case-based educational materials. For consistency, the review focused exclusively on educational resources generated in textual format, such as clinical cases or vignettes, case-based questions, and simulation scenarios. Studies involving the generation of images, audio, or video were therefore excluded. Studies were also excluded if they focused on non-GenAI technologies (eg, rule-based chatbots, expert systems, predictive or classification models without text-generation capabilities), noneducational apps, or educational apps unrelated to case-based material generation.

#### Context

The context of interest was HPE, including medicine, nursing, dentistry, pharmacy, physician assistant or associate education, physiotherapy, occupational therapy, speech and language therapy, dietetics, nutrition, paramedicine, midwifery, and other allied health disciplines.

#### Types of Evidence Sources

Full-text, peer-reviewed original research articles were included to capture empirical evidence on the use of generative LLMs to create case-based educational materials in HPE. Conference papers were also included when sufficient methodological detail was provided. Studies published in English between January 1, 2017, and May 10, 2026, were eligible for inclusion. The start date was selected to capture literature published following the introduction of transformer-based architectures that underpin contemporary LLMs. Only studies available in English were included. The following publication types were excluded: review articles, study protocols, preprints, conference abstracts, commentaries, editorials, letters, book chapters, and nonscholarly sources (eg, blogs and media articles).

### Information Sources

A comprehensive literature search was conducted in 7 electronic bibliographic databases: PubMed, Embase, Scopus, Web of Science, CINAHL, ERIC, and IEEE Xplore. Searches were conducted on May 10, 2026, and covered studies published from January 1, 2017, onwards. All retrieved records were imported into Rayyan (Rayyan Systems Inc), a web-based systematic review management tool, for screening and study selection [22]. Duplicate records were identified using Rayyan’s automated duplicate detection function and subsequently verified through manual review by 1 author (ZZ).

### Search

The search strategy was initially developed by reviewers EL and ZZ and subsequently refined by the review team. It combined controlled vocabulary terms adapted to the database (eg, MeSH and Emtree terms) with free-text keywords, related to 3 concepts: (1) GenAI and LLMs, (2) case-based educational materials, and (3) HPE. The search strategy was iteratively refined in PubMed, and search terms and syntax were subsequently adapted to the indexing and search functionalities of each database. An initial search was conducted on September 8, 2025, and updated on May 10, 2026, following expansion of the search strategy and database coverage. Corresponding authors were contacted by authors (ZZ and ST) when full-text articles could not be obtained through institutional access. The complete database-specific search strategies are provided in Multimedia Appendix 1. Reporting of the search strategy followed the PRISMA-S (Preferred Reporting Items for Systematic Reviews and Meta-Analyses Literature Search Extension) guidelines [23] (Checklist 2).

### Selection of Sources of Evidence

Study selection was conducted using Rayyan in 2 stages: (1) title and abstract screening and (2) full-text review. At each stage, records were assessed independently by 2 reviewers (ZZ, AS, or ST) against the predefined eligibility criteria. Discrepancies were resolved through discussion and consensus. A third reviewer (RT) was consulted in 2 cases where consensus could not be reached.

### Data Charting Process

A structured data charting form was developed in Microsoft Excel to extract relevant information from the included studies. The form was piloted through independent data extraction by 2 reviewers (ZZ and AS) on 3 randomly selected studies and subsequently refined to improve clarity, consistency, and completeness [24]. Data charting was then performed by 1 reviewer (ZZ, AS, or ST) and independently verified by a second reviewer (ZZ, AS, or ST) [24]. Any discrepancies were resolved through discussion and consensus.

### Data Items

Data charted from the included studies [25-96] comprised bibliographic characteristics (authors, title, year, journal, and country); educational context (health profession and learner level); study characteristics (study type and objectives); characteristics of the LLM application (task performed by the LLM, model used, input data or reference materials, prompting strategy, prompt availability, and human oversight); characteristics of the generated case-based materials (type of material and sample size); evaluation approaches (evaluation methods and criteria); and study outcomes, including key findings and reported challenges or limitations. The complete data charting form is provided in Multimedia Appendix 2.

### Synthesis of Results

Charted data were synthesized using descriptive statistics and narrative synthesis. Quantitative data were summarized using frequencies and percentages where appropriate. Findings were organized to address the review objectives and research questions, including (1) characteristics of the included studies [25-96], (2) technical and methodological approaches to LLM-based case generation, (3) evaluation methods and criteria used to assess generated materials, and (4) key findings and reported challenges. Results are presented using tables, narrative summaries, and visual evidence-mapping approaches, including a geographic distribution map and an evidence gap map, to facilitate comparison across studies and identify patterns and gaps in the literature.

## Results

### Selection of Sources of Evidence

A total of 2777 records were identified through 7 databases and citation tracking. After removing 1497 duplicate records, 1280 unique records were screened by title and abstract, with 120 retained for full-text review. Of these, 110 were successfully retrieved and assessed for eligibility, leading to the exclusion of 39 full-text articles. Reasons for exclusion included the absence of LLM-based case generation (n=27), insufficient methodological detail (n=4), non-English publications (n=4), lack of LLM use (n=1), commentary articles (n=1), study protocols (n=1), and framework development papers (n=1). One additional study was identified through citation searching and met the inclusion criteria. Ultimately, a total of 72 studies [25-96] were included in the final synthesis (Figure 1). Detailed charted information for all included studies is provided in Multimedia Appendix 2.

**Figure 1.**
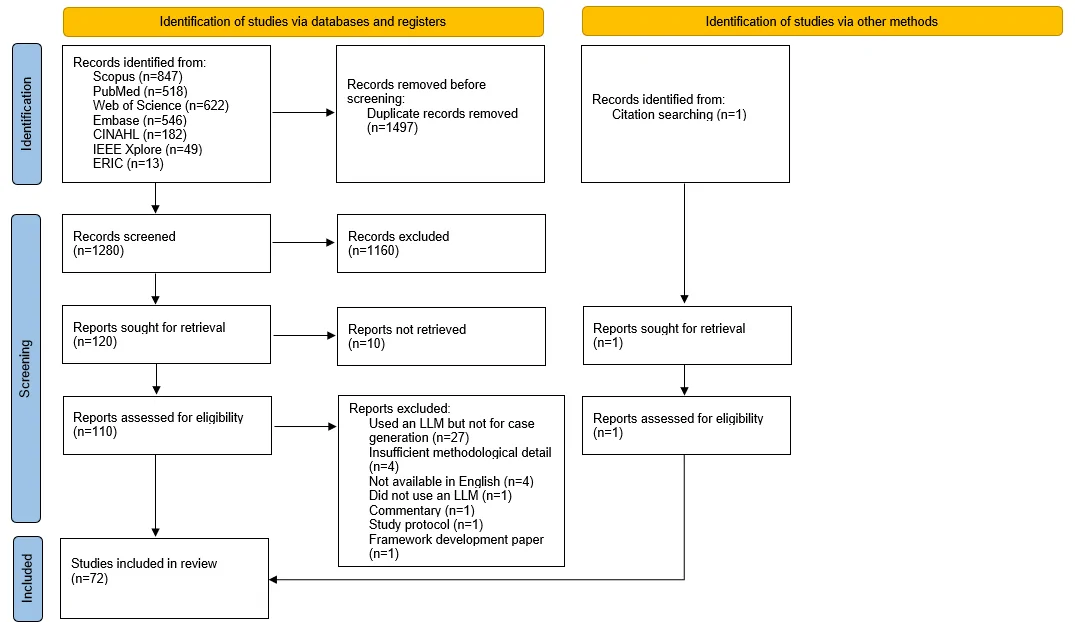
PRISMA (Preferred Reporting Items for Systematic Reviews and Meta-Analyses) flow diagram showing the identification, screening, and inclusion of studies using LLMs to generate case-based materials for HPE. HPE: health professions education; LLM: large language model.

### Characteristics of Sources of Evidence

Most studies were published in 2024‐2025 (53/72, 74%), although an additional 14 studies [25-38] had already been published in 2026 by the beginning of May, highlighting the continued expansion of this field. The included studies most frequently originated from the United States (19/72, 26%) [27,35,44,48,53-55,57,58,60,62,63,74,81,83,87,88,90,95] and Türkiye (10/72, 14%) [28,38,42,47,49,65,72,73,76,93] (Table 1). Geographically, the evidence was concentrated in Asia (35/72, 49%) [26,28,30,31,33,36,38,41,42,45-47,49,50,52,56,65,67-73,76,80,82,84-86,89,91,93,94], and North America (27/72, 37%) [27,35,37,40,44,48,51,53-55,57,58,60-63,66,74,75,78,81,83,87,88,90,95,96], with fewer studies originating from Europe (10/72, 14%) [25,29,31,34,39,43,64,77,79,92], Africa (3/72, 4%) [31,32,43], and South America (1/72, 1%) [59], while no studies were identified from Oceania (Figure 2).

**Table 1.** Characteristics of included studies [25-96] examining the use of LLMs^a^ for the generation of case-based materials for HPE^b^ (N=72).

Characteristics and categories	Studies, n (%)	Article references
Year of publication		
2026	14 (19)	[25-38]
2025	32 (44)	[39-70]
2024	21 (29)	[71-91]
2023	5 (7)	[92-96]
Country		
United States	19 (26)	[27,35,44,48,53-55,57,58,60,62,63,74,81,83,87,88,90,95]
Türkiye	10 (14)	[28,38,42,47,49,65,72,73,76,93]
Canada	5 (7)	[37,61,66,75,96]
India	5 (7)	[26,45,82,84,94]
China	4 (5)	[30,50,52,69]
Kingdom of Bahrain	4 (5)	[31,70,85,91]
Saudi Arabia	4 (5)	[31,41,56,80]
United Kingdom	4 (5)	[31,39,43,77]
Egypt	3 (4)	[31,32,43]
Japan	3 (4)	[67,86,89]
Mexico	2 (3)	[51,78]
Republic of Korea	2 (3)	[33,68]
Spain	2 (3)	[29,34]
Other: Brazil, France, Greece, Grenada, Iran, Switzerland, Italy, Thailand, the Netherlands, United Arab Emirates	9 (12)	[25,36,40,46,59,64,71,79,92]
Study type		
Descriptive or explorative study	10 (14)	[34,59,78,82,84,88,92-95]
Evaluation study (no comparison)	30 (42)	[25,26,31,33,35,36,40,43,44,46,51,53-55,57,58,61,62,68,69,71,74,76,79,81,83,86,87,89,90]
Comparison study: LLM ^a^versus Expert	15 (21)	[30,37,41,45,48,49,52,63,64,66,67,72,75,77,96]
Comparison study: LLM versus LLM	13 (18)	[27-29,38,39,42,50,56,60,70,80,85,91]
Comparison study: LLM versus LLM or other method versus Expert	4 (5)	[32,47,65,73]
Health profession		
Medicine	59 (82)	[25,28-31,34,36-45,47-54,56-58,61,63-66,69-86,88-96]
Nursing	7 (10)	[33,35,46,60,62,67,87]
Pharmacy	2 (3)	[32,55]
Other: dentistry, interprofessional health care, occupational therapy, physiotherapy, respiratory therapy, and paramedicine	5 (9)	[26,27,59,68,96]
Student level		
Undergraduate	42 (58)	[25,26,28-30,32,36-42,45,47,49,51,53-56,58,60-62,65,67,69,70,72,73,75-79,81,83-85,87,91,95]
Postgraduate	8 (11)	[44,48,63,66,70,79,88,89]
Continuing education	1 (1)	[71]
Not specified	22 (30)	[27,31,33-35,43,46,50,52,57,59,64,68,74,80,82,86,90,92-94,96]
Type of case-based materials		
Case-based MCQ^c^	28 (39)	[29,36,39,42,44,45,47-49,55,56,58,64-66,69,70,73,74,76,79-85,93]
Clinical vignette or case	22 (30)	[26,30,31,37,40,41,50,53,57,59,67,68,70-72,78,86,89-92,94]
Simulation scenario	18 (25)	[27,28,32-35,46,51,52,54,60,62,63,77,87,88,95,96]
SCT^d^ or variant	4 (5)	[38,43,61,75]
OSCE^e^, OSPE^f^	3 (4)	[25,70,85]
Short answer question	2 (3)	[70,85]

a LLMs: large language models.

b HPE: health professions education.

c MCQ: multiple-choice question.

d SCT: Script Concordance Test.

e OSCE: Objective Structured Clinical Examination.

f OSPE: Objective Structured Practical Examination.

**Figure 2.**
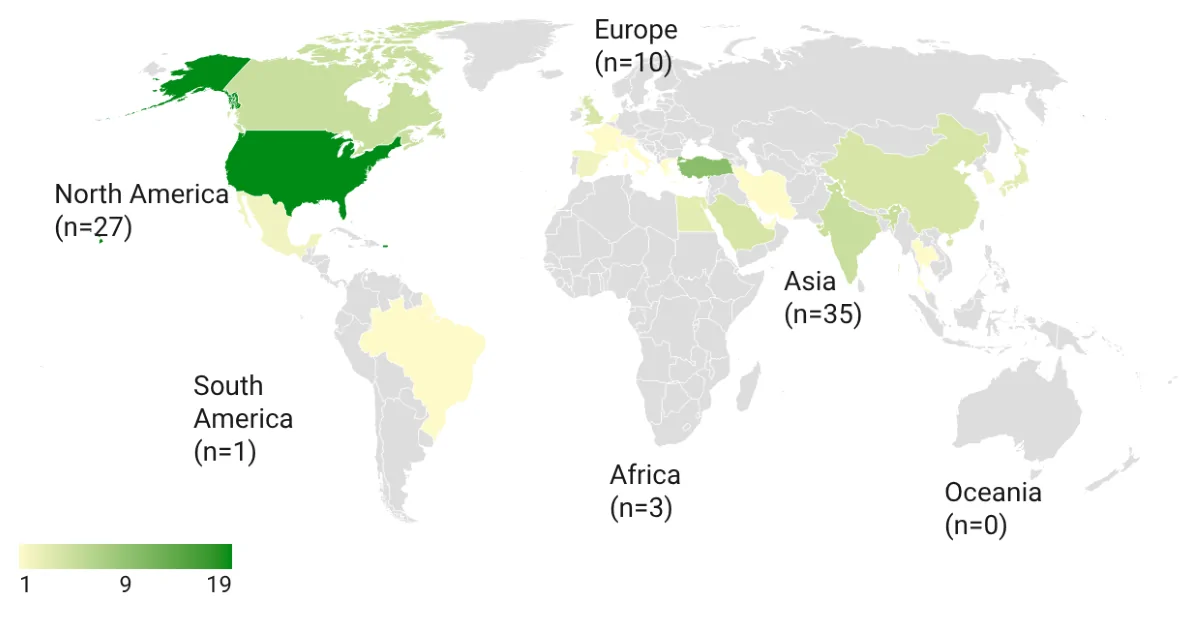
Geographic distribution of studies using LLM to generate case-based educational materials in health professions education by continent. Studies involving multiple countries were counted in each relevant continent; therefore, continent totals exceed the total number of included studies.

The most frequent study design was LLM evaluation studies without comparison groups (30/72, 42%) [25,26,31,33,35,36,40,43,44,46,51,53-55,57,58,61,62,68,69,71,74,76,79,81,83,86,87,89,90]. Comparative approaches were also common and included comparisons between LLM-generated and expert-generated materials (15/72, 21%) [30,37,41,45,48,49,52,63,64,66,67,72,75,77,96], between different LLMs (13/72, 18%) [27-29,38,39,42,50,56,60,70,80,85,91], or between LLMs or other generation methods and expert-generated materials (4/72, 5%) [32,47,65,73]. Descriptive or exploratory studies accounted for 10/72 (14%) [34,59,78,82,84,88,92-95] of the included publications (Table 1).

Case-based MCQs were the most frequently generated materials (28/72, 39%) [29,36,39,42,44,45,47-49,55,56,58,64-66,69,70,73,74,76,79-85,93], followed by clinical cases or vignettes (22/72, 30%) [26,30,31,37,40,41,50,53,57,59,67,68,70-72,78,86,89-92,94] and simulation scenarios (18/72, 25%) [27,28,32-35,46,51,52,54,60,62,63,77,87,88,95,96]. Medical education was the predominant focus (59/72, 82%) [25,28-31,34,36-45,47-54,56-58,61,63-66,69-86,88-96], and undergraduate students were the most commonly targeted population (42/72, 58%) [25,26,28-30,32,36-42,45,47,49,51,53-56,58,60-62,65,67,69,70,72,73,75-79,81,83-85,87,91,95], although student level was not specified in nearly one-third of studies (Table 1). The evidence gap map (Figure 3) highlighted substantial variation across health professions. Medical education studies covered all material types, particularly case-based questions, whereas nursing studies (7/72, 10%) [33,35,46,60,62,67,87] were concentrated on simulation scenarios. Pharmacy studies (2/72, 3%) [32,55] generated case-based questions and simulation scenarios, while studies involving other health professions (5/72, 7%) [26,27,59,68,96] predominantly generated clinical cases or vignettes. Few studies targeted postgraduate or continuing education learners, revealing important gaps in the current evidence base.

**Figure 3.**
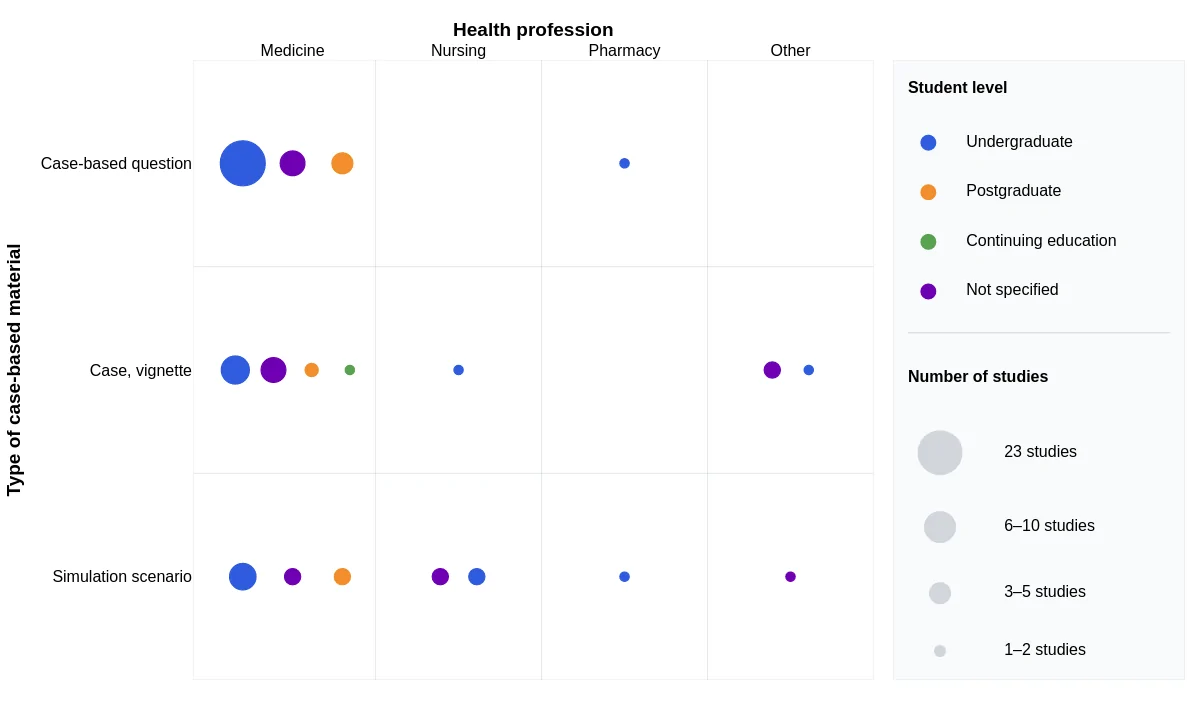
Evidence gap map of studies using large language models to generate case-based educational materials in HPE. Bubble plot summarizing the distribution of included studies [25-96] according to the type of generated case-based material and target health profession. Generated materials were grouped into 3 categories: case-based questions, including case-based MCQs, short-answer questions, script concordance tests (SCTs), and variants; cases or vignettes; and simulation scenarios, including OSCE and OSPE scenarios. Bubble size is proportional to the number of included studies within each category, while bubble color indicates student level. HPE: health professions education; MCQs: multiple-choice questions; OSCE: Objective Structured Clinical Examination; OSPE: Objective Structured Practical Examination.

### Synthesis of Results

#### Use of LLMs for Case-Based Material Generation

Generative Pretrained Transformer-4 (GPT-4) (OpenAI) family models were the most frequently used (40/72, 55%) [25,26,28,29,32-34,36,37,39,42,43,45-49,51-56,60,61,63-66,69-71,73,77,80,81,84,86,89,90], followed by GPT-3.5 family models (21/72, 29%) [29,39,40,44,57,65,67,68,72,74-76,78-80,83,85,91,92,95,96]. Other commonly used models included Bard and Gemini (Google AI) (13/72, 18%) [27,28,32,38,39,41,42,47,56,58,65,80,91], GPT models with unspecified versions (9/72, 12%) [27,31,35,59,62,82,87,88,94], Claude (Anthropic) (7/72, 10%) [27,28,32,47,56,60,85], and DeepSeek (5/72, 7%) [28,30,32,56,70], while several other models were reported only occasionally.

Although several studies cited guidelines to follow, only a few provided these resources directly to the LLM (7/72, 10%) [27,50,56,65,80,85,91], and most studies relied on prompt-only generation (59/72, 82%) [26-32,34,35,37-40,42,44-46,49-54,56,58,60-66,68,69,71-88,90-96]. The majority of studies published their prompts in full or in part (68/72, 94%) [25-56,58-66,68-72,74-93,95,96], and all studies used basic instructional and contextual prompting. Among more advanced prompting techniques, output formatting or template-based prompting was the most common (57/72, 79%) [25,26,28-38,42-63,65,68-73,75-77,79-81,83,85,86,89,90,92,93,95,96], whereas role prompting (22/72, 30%) [25,31-33,38,39,42,43,47,51,52,59-61,66,69-71,75,77,86,89], prompt chaining (15/72, 21%) [25,33,40,41,48,52,57,59,60,62,70,74,86,92,96], and few-shot prompting (6/72, 8%) [25,33,47,48,59,89] were used less frequently. Refinement strategies were reported in several studies, most commonly prompt refinement (25/72, 35%) [27,29,32,33,36,37,40,43,45,58,60,62,63,66,68,69,72,78,79,83,85,88,92,95,96], followed by output refinement by experts or authors (16/72, 22%) [26,35-37,44-47,49,55,57,62,63,65,77,96] and LLM self-refinement (5/72, 7%) [33,52,57,72,86]. The quantity of generated materials varied. Most studies generated ≤10 outputs (29/72, 40%) [26,30,33,35,41,43,46,49,51,58,59,62,64,68,71,74-78,82-84,87,88,93-96] or between 11 and 100 outputs (25/72, 35%) [25,27,28,36-38,44,45,47,48,52,53,55,57,61,63,65,66,69,72,73,79,86,91,92], whereas 14 studies (19%) [29,32,34,39,40,42,50,54,56,80,81,85,89,90] generated more than 100 outputs (Table 2).

**Table 2.** LLM^a^ generation approaches and technical characteristics for case-based material generation in health professions education (N=72).

Characteristics and categories	Studies, n (%)	Article references
Model used		
GPT-4^b^ family (OpenAI)	40 (55)	[25,26,28,29,32-34,36,37,39,42,43,45-49,51-56,60,61,63-66,69-71,73,77,80,81,84,86,89,90]
GPT-3.5 family (OpenAI)	21 (29)	[29,39,40,44,57,65,67,68,72,74-76,78-80,83,85,91,92,95,96]
Bard Gemini (Google AI)	13 (18)	[27,28,32,38,39,41,42,47,56,58,65,80,91]
GPT unspecified version (OpenAI)	9 (12)	[27,31,35,59,62,82,87,88,94]
Claude (Anthropic)	7 (10)	[27,28,32,47,56,60,85]
DeepSeek (DeepSeek AI)	5 (7)	[28,30,32,56,70]
Bing Copilot (Microsoft)	4 (5)	[27,39,60,80]
GPT-5 family (OpenAI)	2 (3)	[36,38]
Other (models used in ≤2 articles: Baichuan, Ernie, GPT o1 and o3, Grok, Mixtral, PaLM, Poe, Qwen)	7 (10)	[27,50,56,65,80,85,91]
Input data or reference material		
None (prompt-only)	59 (82)	[26-32,34,35,37-40,42,44-46,49-54,56,58,60-66,68,69,71-88,90-96]
Examples or prior cases	6 (8)	[25,33,41,47,59,89]
Textbook chapters or guidelines	7 (10)	[25,36,43,48,55,57,70]
Prompt publication		
Yes	62 (86)	[25-40,42-56,58,60-63,68,70,72,74-93,95,96]
Partly (extracts)	6 (8)	[41,59,64,65,69,71]
No	4 (5)	[57,67,73,94]
Prompting techniques		
Basic		
Instructional	71 (99)	[25-66,68-96]
Contextual	71 (99)	[25-66,68-96]
Output format Template	57 (79)	[25,26,28-38,42-63,65,68-73,75-77,79-81,83,85,86,89,90,92,93,95,96]
Advanced		
Role	22 (30)	[25,31-33,38,39,42,43,47,51,52,59-61,66,69-71,75,77,86,89]
Prompt chaining	15 (21)	[25,33,40,41,48,52,57,59,60,62,70,74,86,92,96]
One-shot Few-shot	6 (8)	[25,33,47,48,59,89]
Refinement process		
Prompt refinement	25 (35)	[27,29,32,33,36,37,40,43,45,58,60,62,63,66,68,69,72,78,79,83,85,88,92,95,96]
Output refinement	16 (22)	[26,35-37,44-47,49,55,57,62,63,65,77,96]
Self-refinement (by the LLM)^a^	5 (7)	[33,52,57,72,86]
Sample size (materials generated)		
≤10 outputs	29 (45)	[26,30,33,35,41,43,46,49,51,58,59,62,64,68,71,74-78,82-84,87,88,93-96]
11‐100 outputs	25 (35)	[25,27,28,36-38,44,45,47,48,52,53,55,57,61,63,65,66,69,72,73,79,86,91,92]
>100 outputs	14 (19)	[29,32,34,39,40,42,50,54,56,80,81,85,89,90]
Not specified	4 (5)	[31,60,67,70]

a LLM: large language model.

b GPT-4: Generative Pretrained Transformer-4.

#### Evaluation of LLM-Generated Case-Based Materials

Most studies used expert review as the primary evaluation method (56/72, 78%) [25-33,35-39,41-49,51-58,60,62,63,65-73,75-77,79-81,83,85-87,89,91,96], while fewer incorporated student assessment (25/72, 35%). Statistical analyses for bias testing were uncommon (4/72, 5%), and 10/72 studies (14%) were exploratory without a formal evaluation.

Regarding the volume of evaluated materials, 30% (22/72) of studies assessed ≤10 outputs, 35% (25/72) [26,28,30,32,36,42,44,45,47-49,51,52,55,61-67,72,76,77,79] evaluated 11‐100 outputs, and 15% (11/72) [29,32,34,40,50,54,56,80,85,89,90] evaluated >100 outputs, while 7% (5/72) [31,57,60,67,70] did not report the number of materials evaluated. Among studies using expert review, most involved 10 or fewer experts (42/72, 58%) [25,27,29,31,33-35,37-41,43,46,50,53,54,56-60,68-71,73-75,78,80-96], whereas only 7/72 (10%) [63,71,73,77,86,87,96] engaged 11‐100 experts and 1/72 (1%) [75] involved more than 100 experts; 22% (16/72) [34,40,50,59,61,64,74,78,82,84,88,90,92-95] did not include expert evaluation and 8% (6/72) [30,52,57,62,72,76] did not specify the number of experts involved. Student assessment most commonly involved 11‐100 students (16/72, 22%) [26,28,36,42,44,48,51,55,61,63-66,72,76,79], while only a few studies included larger student samples (Table 3).

**Table 3.** Evaluation methods of LLM^a^-generated case-based materials for health profession education (N=72).

Category	Studies, n (%)	Article references
Evaluation method		
Expert review	56 (78)	[25-33,35-39,41-49,51-58,60,62,63,65-73,75-77,79-81,83,85-87,89,91,96]
Student assessment	25 (35)	[26,28,30,32,36,42,44,45,47-49,51,52,55,61-67,72,76,77,79]
Statistical analysis	4 (5)	[34,40,50,90]
No formal evaluation (exploratory or descriptive)	10 (14)	[59,74,78,82,84,88,92-95]
Sample size (materials evaluated)		
≤10 outputs	22 (30)	[26,30,33,35,41,43,46,49,51,58,59,62,64,68,71,73,75-77,83,87,96]
11‐100 outputs	25 (35)	[25,27,28,36-39,42,44,45,47,48,52,53,55,61,63,65,66,69,72,79,81,86,91]
>100 outputs	11 (15)	[29,32,34,40,50,54,56,80,85,89,90]
Not specified	5 (7)	[31,57,60,67,70]
Number of experts involved in evaluation		
≤10 experts	42 (58)	[25-29,31-33,35-39,41-49,51,53-56,58,60,65-70,79-81,83,85,89,91]
11‐100 experts	7 (10)	[63,71,73,77,86,87,96]
>100 experts	1 (1)	[75]
Not specified	6 (8)	[30,52,57,62,72,76]
No expert evaluation	16 (22)	[34,40,50,59,61,64,74,78,82,84,88,90,92-95]
Number of students involved in evaluation		
≤10 students	1 (1)	[67]
11‐100 students	16 (22)	[26,28,36,42,44,48,51,55,61,63-66,72,76,79]
101‐300 students	5 (7)	[30,32,45,47,77]
>300	1 (1)	[49]
Not specified	2 (3)	[52,62]
No student assessment	47 (72)	[25,27,29,31,33-35,37-41,43,46,50,53,54,56-60,68-71,73-75,78,80-96]

a LLM: large language model.

Across the 62 studies [25-58,60-73,75-77,79-81,83,85-87,89-91,96] that conducted an evaluation, metrics clustered into a few major groups, but their operationalization varied widely. In expert-based evaluations, the most frequently assessed dimensions were clinical accuracy (38/62, 61%) [25-29,32,36,37,39,41-46,49,51,53,54,56-58,62,69-71,73,76,80,81,83,85-87,89,91,96], educational alignment (31/62, 50%) [25-28,30-32,35-39,41-46,51,52,55,56,60,68,70,76,79-81,85,86], and clinical realism (27/62, 44%) [25,27,28,31-33,41-46,50,52,60,62,68-71,75,77,79,86,87], with notable variation in how each was defined (eg, free-text qualitative judgments vs structured Likert ratings). In student-based evaluations, psychometric analyses were the most common, particularly discrimination indices (11/62, 18%) [36,42,45,47-49,55,66,76,77,79] and difficulty indices (10/62, 16%) [36,42,45,48,49,55,65,76,77,79]. Student perceptions were also frequently evaluated through Likert-scale surveys (11/62, 18%) [26,28,30,32,51,52,61,63,64,67,72], whereas measures of student performance, such as correct response rates or pre-post comparisons, were less common. Other evaluation approaches included assessments of bias (8/62, 13%) [27,29,40,50,53,60,70,90], dataset validity (1/62, 2%) [34], and practical considerations such as time, cost, consistency, and workload associated with case generation (6/62, 10%) [30,39,47,67,77,96] (Table 4).

**Table 4.** Evaluation criteria of LLM^a^-generated case-based materials for health profession education, in studies reporting an evaluation (N=62)^b^.

Criteria	Studies, n (%)	Articles and examples
Expert review		
Qualitative (Independent expert review, group consensus, and focus group discussion)		
Clinical accuracy	11 (18)	[42,44-46,51,62,66,86,87,91,96] [42]: “technical accuracy”; [44]: “clinical appropriateness”; [46]: “clinical accuracy,” “alignment with current medical guidelines and best practices”;[86,87,96]: free-text responses noting the inaccuracies
Educational alignment	8 (13)	[30,42,44-46,51,60,68] [46]: “educational effectiveness, relevance to nursing competencies, learning outcomes”
Clinical realism	7 (11)	[42,44-46,60,62,68] [42]:“clinical relevance”; [44]: “ethical realism, and contextual relevance”; [46]: “scenario realism”
Completeness, sufficiency of information	4 (6)	[60,66,68,91]
Bias	2 (3)	[27]: “allow evaluators to identify content perceived as biased or stereotypical of the patient populations”; [60]
Clarity, coherence, and language quality	1 (2)	[91]: “vague instructions for drug administration,” “use of medical terms less likely understood by patient”
Format, construction quality	1 (2)	[66]: “quality of the stem construction, plausibility of the distractors”
Open-ended feedback	18 (29)	[25-28,31,35-37,43,45,46,55,60,69,75,86,87,96] [28]: “what do you think about using such scenarios in clinical education?”; [75]: “advantages and drawbacks of the presented SCTs^c^”; [87]: “what surprised you about the information ChatGPT presented?”
Quantitative (Likert scales, binary (yes, no))		
Clinical accuracy	28 (45)	[25-29,32,36,37,39,41,43,49,53,54,56-58,69-71,73,76,80,81,83,85,86,89]
Educational alignment	26 (42)	[25-28,31,32,35-39,41,43,45,52,55,56,68-70,76,79-81,85,86]
Format, construction quality	23 (37)	[25,28,29,31-33,35,37-39,49,56,58,69,70,73,75,76,79-81,83,85]
Completeness, sufficiency of information	22 (35)	[25,28,29,32,33,35,36,38,41,50,53,57,58,68-70,73,76,79,85,86,89]
Clinical realism	21 (34)	[25,27,28,31-33,37,38,41,43,50,52,68-71,75,77,79,86,87]
Clarity, coherence, language quality	21 (34)	[25,28,32,33,36,37,39,41,43,45,49,58,69-71,73,75,76,79,86,89]
Difficulty, challenge appropriateness	19 (31)	[28,29,37,38,41,52,56,58,63,69-71,73,75,77,80,81,83,86]
Engagement, reflection	11 (18)	[25,28,36,37,41,52,58,68-71]
Usability, exam readiness	10 (16)	[25,27,28,31,36,38,47,50,55,89]
Content validity	3 (5)	[33,39]: “Item Content Validity Index” [55]; “Content Validity Ratio”
Blinded attribution (human vs LLM)	2 (3)	[63,75]
Feasibility	2 (3)	[25,37]
Originality	2 (3)	[25,28]
Bias	2 (3)	[53]: “possibility of demographic bias”; [70]: “cultural and contextual sensitivity; diversity and representation; ethical and professional considerations”
Overall quality or rating	5 (8)	[25,26,28,53,96]
Other	4 (6)	[31]: “human oversight,” “importance of local adaptation”; [71]: “which group the cases were most suitable for: medical students, medical residents, health professionals, or all of the above”; [56,80]: “variety (does it repeat previous responses?)”
Student assessment		
Psychometric analysis		
Discrimination index	11 (18)	[36,42,45,47-49,55,66,76,77,79]
Difficulty index	10 (16)	[36,42,45,48,49,55,65,76,77,79]
Distractor effectiveness	4 (6)	[36,45,47,76]
Reliability	4 (6)	[42,79]: Cronbach α; [45,55]: KR-20 reliability coefficient
Other	1 (2)	[55]: “passing standard (Angoff method),” item response theory and Rasch analysis”
Student performance		
Performance tracking during intervention or pre-post intervention	3 (5)	[42,44,67]
Correct response rate	4 (6)	[47,48,55,66]
Group-level performance comparison (between student levels or specialties)	3 (5)	[42,48,79]
Student evaluation		
Likert scale survey	11 (18)	[26,28,30,32,51,52,61,63,64,67,72] [51]: “scenario construction (clarity, realism of the case), development or execution (interactivity and simulator responses), relevance (usefulness for clinical training, appropriate level of challenge)”; [52]: “student engagement”; [61]: “perceived difficulty”; [72]: “clarity, spelling, contradictions, enjoyment, clinical reasoning, keyword generation, research question suitability, overall liking, EBM learning support, need for correction”
Blinded attribution (Human vs LLM)	5 (8)	[28,48,52,61,63]
Other		
—^d^	6 (10)	[30,47,67,77,96]: time to produce cases; [39]: “consistency between chatbot outputs across the 3 researchers (exact, similar or different),” “assessment of bias or preference in the correct answer allocation”; [67]: “perceived burden of case creation”; [77]: “financial cost of drafting GPT-assisted vs standard cases”
Statistical analysis		
Bias analysis		
—	5 (8)	[27]: descriptive statistics of sociodemographic data (gender, race, ethnicity, English proficiency, sexual orientation, marital status, employment, household income, source of payment for health services, and living situation); [29]: *χ*² tests to compare demographic and clinical content features across MCQs; [40,50,90]: epidemiological concordance (comparison of distributions against actual prevalence data); [40]: “compare changes in ethnoracial representation between March and May 2024 and differences between initial prompting and self-prompting”; [90]: “impact of geographic setting or using debiasing strategies”
Dataset validity		
—	1 (2)	[34]: distribution and relationships between variables of age, gender, occupation, reason for consultation, medical history, vital signs and diagnosis

a LLM: large language model.

b Studies could evaluate the same criterion using both qualitative and quantitative approaches. Consequently, a study may appear in more than 1 row for a given evaluation criterion. Counts reported in the narrative synthesis represent unique studies and therefore may differ from the sum of qualitative and quantitative counts shown in this table.

c SCTs: Script Concordance Tests.

d Not available.

#### Key Findings and Challenges Identified in the Included Studies

##### Clinical Accuracy

LLM-generated materials were often assessed as clinically accurate by experts [25,28,31,39,42,43,46,53,56,57,68,73,83,85,86,89], particularly for common conditions [54] and structured scenarios [25,43]. For example, Rao et al [53] reported a mean scientific accuracy score of 4.45/5, Saad et al [83] found that 80% of items met medical accuracy criteria, and Ananthanarayanan [57] reported medical accuracy scores ranging from 9.59 to 10.0 out of 10. At the same time, inaccuracies and missing or contradictory clinical information were frequently reported, including hallucinations [32,69], diagnostic inconsistencies and inappropriate investigations [46,63,69,86,87,89], pharmacotherapeutic errors and inaccurate treatment recommendations [32,46,58,62,76,83,89,91,92], and incomplete patient histories or missing clinical details [25-27,29,33,35,46,60,63,69,74,79,86,87,89,92,96]. These findings show that while LLMs can generate clinically acceptable drafts, expert validation and revision are still required to ensure accuracy.

##### Structural Quality

Structural quality, including clarity, language quality, coherence, and adherence to construction rules, was frequently examined. Several studies reported clear wording in LLM-generated materials [25,28,32,36,37,39,41,43,73,75,76]. Language quality and medical terminology accuracy in non-English outputs varied, with Turkish-language scenarios praised for their linguistic precision and understandability [28], whereas Japanese-language vignettes exhibited occasional issues with terminology use, grammatical correctness, and language consistency, including sporadic insertion of nontarget languages [86,89]. Reviewers also highlighted concise case descriptions and vignettes that included the information necessary to support learning [36,38,69,71]. However, some studies identified inconsistencies between case narratives and associated questions [69], excessive length despite instructions to be concise [56], unnecessary detail [55], and occasional coherence problems in medical contexts [28]. Recurring construction problems further limited direct reuse, including positional answer bias and variable item balance [39], multiple, ambiguous, or incorrect answers [32,36,48,49,66,79,85], repetitive or shallow scenarios [80], and pseudovignette construction [37,58,83].

##### Educational Alignment and Pedagogical Quality

Educational alignment was frequently assessed across studies. Many studies showed that LLM-generated materials could appropriately target intended competency levels and learning objectives [25-28,30-32,36-38,41,45,46,52,55,68-70,72,76,85,86]. For example, Arain et al [41] reported higher alignment with learning objectives for LLM-generated vignettes than for faculty-authored versions (mean 3.0 vs 1.25), and Takahashi et al [86] reported a mean “educational usefulness” score of 3.55/5 across 18 vignettes. However, challenges included inconsistent alignment across training stages [85], the need for adaptation to local curricula [31,35,76], some lack of alignment with course objectives [26,45], and pedagogical limitations such as the presence of leading formulations or cues [25,37,41,58,66,69,70,79], as well as a tendency toward low-complexity, recall-level items, insufficiently challenging students [37,41,55,58,66,69,83,85]. Iterative refinement approaches were associated with improved educational alignment comparable to expert-authored scenarios [52].

##### Clinical Realism

Clinical realism was variably achieved. Several studies found that LLM-generated materials were generally realistic, with experts and students rating them as plausible [28,31,38,46,51,68,69,77], although realism appeared to improve with more structured and iterative prompting strategies [25,33] and varied across LLMs [28,60]. Despite generally high ratings for narrative plausibility, one study [32] found that student satisfaction did not align with expert assessments of clinical accuracy and safety, suggesting that realistic narratives may obscure underlying inaccuracies. Common problems were also reported, including less realistic presenting illness timelines or physical examination data, vague terminology, and lack of patient-centered context [27,86]; unrealistic combinations (eg, mismatched names and ethnic backgrounds or unusual patient behavior [92]); and overly generic or stereotypical cases with limited cultural and linguistic relevance [44,45,62,69,75,87]. These limitations were further evidenced when several LLM-generated exam items required exclusion due to unrealistic scenarios [55]. Overall, superficial realism was common, but expert refinement was typically required to achieve realism.

##### Psychometric Analysis and Student Perception

Psychometric analyses showed mixed results. Difficulty indices were frequently acceptable for classroom use [42,48,49,76,77,79]. Comparisons with faculty-authored questions yielded mixed findings, with several studies reporting LLM-generated items to be easier [45,55], whereas Yapici Coşkun et al [65] found LLM-generated questions to be substantially more difficult and less balanced across difficulty levels. Discrimination indices were generally acceptable [36,47,48,77,79], but lower discrimination than faculty-written items was consistently observed in some settings [42,45,66,76]. Distractor effectiveness was frequently suboptimal, with high rates of nonfunctional distractors reported [36,45,47,76]. Student performance analyses showed comparable correct response rates between LLM- and human-authored items [47,48,66], improvements over time during interventions [42,44], and variable group-level performance across student levels and subspecialties [42,48,79]. Furthermore, student surveys generally indicated high engagement and favorable perceptions of LLM-generated materials [26,28,30,32,51,52,72], and blinded attribution evaluations reported difficulty distinguishing LLM- from human-authored materials [48,52,61,63,75].

##### Bias Analysis

Bias issues were explicitly evaluated in a subset of studies [27,40,50,90]. Large-scale analyses showed that generated materials differed substantially from real-world disease prevalence and frequently overrepresented stereotyped groups [90]. Similarly, initial prompting often produced highly stereotyped ethnoracial assignments, while self-prompting altered distributions without restoring epidemiological concordance [40]. Model-specific bias profiles were also observed, including systematic over- or underrepresentation of minority, low-income, and low-education groups across conditions [50]. Bias evaluation of LLM-generated geriatric simulation scenarios revealed limited representation of LGBTQIA + (Lesbian, Gay, Bisexual, Transgender, Queer or Questioning, Intersex, and Asexual) individuals, Indigenous populations, and social determinants of health, with most cases occurring in hospital or primary care office [27]. Other studies reporting on bias noted limited demographic diversity, stereotypical trait-occupation pairings, and culturally incomplete or oversimplified representations [29,53,62,78,92]. Notably, findings from Sridharan et al [70] suggested that incorporating explicit inclusivity criteria into prompt design may support more diverse and culturally responsive content. These studies highlighted a persistent risk of reinforcing social stereotypes and misrepresenting population prevalence in educational materials, potentially affecting clinical training and increasing automation bias.

##### Comparison of Model Performance

Multiple studies directly compared several LLMs [27,28,32,38,39,42,47,56,60,70,80,85,91], with results varying across models and studies. In case-based question generation tasks, Abouzeid et al [39] found that Gemini produced more consistent items, while ChatGPT-4 (OpenAI) generated greater variability in question content and structure. Robison et al [60] similarly reported distinct strengths, with ChatGPT excelling in detail-rich narratives and structured approaches, Claude demonstrating strong communication and diversity, and Copilot providing valuable educational resources but requiring greater refinement for accuracy and depth. In professionalism and ethical decision-making item generation, İş-Kara and Kıyak [38] found that Gemini 2.5 Pro achieved higher global suitability rates and checklist scores than ChatGPT-5 Thinking, producing more realistic vignettes and more consistently appropriate levels of ambiguity for learners. By contrast, Başaranoğlu et al [42] found no significant difference in technical accuracy between urology MCQs generated by ChatGPT-4 and Gemini 1.5. Also in urology, Szabó and Laein [56] assessed 13 models and found Claude-3.5 Sonnet achieved the highest scores for correctness, relevance, complexity, and variety; though all models showed systematic content imbalance through overrepresentation or omission of key concepts.

Comparisons involving clinical cases and simulation scenarios yielded similarly mixed findings. In gross anatomy, ChatGPT-4 achieved the highest scores, followed by Gemini and ChatGPT-3.5 variants, while Copilot (Microsoft AI) and PaLM 2 (Google) showed high repetition rates and ranked lowest [80]. A comparable pattern was observed in geriatric scenario generation, where ChatGPT achieved the highest overall quality scores, followed by Claude, whereas Grok received the lowest ratings because of incomplete outputs [27]. However, other studies favored different models. Farrag et al [32] found that Gemini achieved the highest overall success rate, Claude achieved the highest clinical accuracy scores, DeepSeek performed best in instructional design quality and clinical reasoning fidelity, whereas GPT-4o showed the lowest performance. Similarly, Öncü et al [28] reported that Claude-3.7 Sonnet produced the highest-quality Turkish-language scenarios, outperforming ChatGPT-4o, Gemini 1.5, and DeepSeek in measures of clarity, realism, educational value, and support for clinical reasoning.

Finally, a subset of studies focused on pharmacotherapy and likewise revealed complementary performance. Güvel et al [47] showed that Claude-3.5 Sonnet produced the highest proportion of error-free pharmacotherapy questions, whereas ChatGPT-4o generated the fewest unusable items. Sridharan and Sequeira [85,91] observed substantial overlap among generated materials, with ChatGPT-3.5 generally providing more detailed explanations and patient-oriented guidance, whereas all models required expert review because of omissions and inconsistencies. More recently, Sridharan and Sivaramakrishnan [70] found that both DeepSeek-V3 and ChatGPT-4.0 generated pedagogically sound materials, but DeepSeek produced more rubric-aligned, contextually rich, and assessment-ready content. Importantly, despite differences in rankings across studies, these findings showed that no model generated case-based materials without identifiable flaws across evaluated domains.

## Discussion

### Summary of Evidence

This scoping review showed that the use of LLMs to generate case-based materials in HPE has expanded rapidly since 2023, predominantly within undergraduate medical education and largely focused on the production of case-based MCQs and clinical vignettes. In the 72 studies [25-96] reviewed, LLMs were mostly used with simple prompts. More advanced strategies, such as few-shot prompting, prompt chaining, or using external knowledge sources, were rarely applied. Evaluation was usually based on small expert reviews, while psychometric testing and student-based validation were less common. Overall, LLM-generated materials were frequently judged to be clinically plausible, structurally clear, and reasonably aligned with educational objectives, particularly after refinement. However, consistent limitations emerged, including factual inaccuracies, construction flaws, superficial clinical realism, pedagogical misalignment with curricula or training level, suboptimal psychometric performance, and recurrent demographic and epidemiological bias. Finally, comparative studies reported varying performance across LLMs, with no model consistently outperforming others. However, methodological differences between studies limit conclusions about which models perform best.

### Comparison With the Literature

Our findings are consistent with previous reviews examining the use of GenAI in HPE. Basil et al [3], in a broad scoping review of 241 studies, identified content creation as the predominant educational application of GenAI, followed by assessment, with medicine and nursing accounting for most published research. They also highlighted generally positive perceptions of GenAI alongside ongoing concerns regarding accuracy, ethical considerations, governance, and the need for more rigorous evaluation of educational outcomes. Similarly, Astbury et al [2] reviewed 23 studies focused on teaching and assessment and found that GenAI was primarily used for learning resource development, assessment, feedback, and simulation, with reported benefits including personalized learning, improved efficiency, and resource use, but persistent concerns regarding accuracy, inconsistent outputs, algorithmic bias, and academic integrity. Our findings reinforce these broader observations while providing a focused and comprehensive synthesis of one important educational application: the generation of case-based educational materials.

A recent review, Kaya et al [20], specifically examined LLM-generated cases. Our findings broadly aligned with their conclusion that LLM-generated cases represent a promising but evolving educational resource, supported by a heterogeneous evidence base. Similar to Kaya et al [20], we found that GPT-based models were the most frequently used LLMs, medicine was the predominant health profession represented, and expert evaluation remained the primary method for assessing generated materials, underscoring the continued need for human oversight before educational implementation. Consistent with their findings, we also observed that prompts were reported in most studies, although prompting approaches varied considerably. However, whereas they primarily described whether the prompt was reported and the use of single versus iterative prompting, our review extends these findings by providing a more granular characterization of generation approaches. In addition to reporting prompt use, we examined the use of reference materials, prompting techniques, and refinement processes, showing that generation predominantly relied on relatively simple prompting strategies with limited adoption of more advanced prompt engineering. Furthermore, while the authors described quality evaluation in broad categories, such as overall case quality and bias, our review also offers a more detailed synthesis of quality assessment by integrating both evaluation methods and findings across multiple quality domains, including clinical accuracy, structural quality, educational alignment, pedagogical quality, realism, psychometric performance, learner perceptions, and bias. This broader perspective provides a more comprehensive understanding of current practices, methodological limitations, and evidence gaps. Finally, our review substantially captures a wider spectrum by synthesizing more than 3 times as many studies, encompassing a broader range of case-based educational materials, including clinical cases, case-based MCQs, Objective Structured Clinical Examination (OSCE) or Objective Structured Practical Examination (OSPE) scenarios, simulation scenarios, script concordance tests (SCTs), and short-answer questions.

### Future Directions

#### From Prompt-Only Generation Toward Robust Generation Pipelines

As shown in our review, most articles relied on relatively simple, prompt-based generation approaches and reported variable output quality that often required expert refinement. These findings underscore the need to move toward more robust generation pipelines. Future research should therefore prioritize structured pipelines that integrate more sophisticated technical strategies, including advanced prompting strategies, grounding in reliable reference sources, and iterative expert oversight.

More advanced prompting strategies, including prompt few-shot, chain-of-thought (CoT), template-based, self-refinement or iterative refinement, and agentic workflows, have the potential to improve the accuracy, clarity, and reliability of LLM outputs [97-100]. For example, among included studies, Qingquan et al [52] reported that iterative refinement improved the quality of LLM-generated clinical scenarios compared with single-step prompting, with performance comparable to or exceeding human-developed cases. Another promising approach is retrieval-augmented generation (RAG). Introduced in 2020, RAG enables LLMs to generate responses based not only on pretrained knowledge, but also on information retrieved from external, trusted sources such as up-to-date clinical guidelines, textbooks, or medical literature [101,102]. A growing body of evidence has shown that RAG-based systems can enhance LLMs for health care-related tasks (eg, medical question answering, decision support, and summarization), particularly in terms of accuracy, relevance, and reliability [103-106]. Although RAG was rarely used in the studies included in our review, its potential was illustrated by Ananthanarayanan [57], who combined RAG with iterative AI-feedback refinement to generate diagnostic cases. Their pipeline retrieved relevant medical knowledge before generation and iteratively refined outputs using an AI critic, producing cases with excellent medical accuracy.

A simpler alternative to RAG is the use of customized GPTs [107], personalized versions of GPT, configured with precise instructions and domain-specific reference documents to perform targeted tasks. A few studies included in our review adopted this approach. Shultz et al [55] developed a custom GPT using pharmacotherapy textbook chapters and National Board of Medical Examiners (NBME) item-writing guidelines to generate pharmacy examination questions. Abouzeid and Sallam [43] developed a customized GPT for SCT generation by incorporating script theory, relevant literature, and expert-derived scoring keys, whereas Abouzeid et al [31] designed a “Problem-based learning (PBL) Case Builder” that integrates curricular parameters and structured pedagogical guidance to generate PBL cases. Similarly, Zouakia et al [25] used a customized GPT enriched with domain-specific reference documents to generate OSCE stations in digital health, demonstrating that combining a knowledge base with structured prompting improved the quality of generated content.

Finally, robust generation pipelines may benefit from iterative expert-in-the-loop workflows. Incorporating expert feedback across multiple refinement rounds could also improve LLM outputs, aligning with established medical item-development practices and reflecting the level of oversight required in high-stakes contexts [108]. Among included studies [25-96], Coşkun et al [72] described a workflow in which experts reviewed LLM-generated clinical vignettes, identified cases that were insufficiently specific or pedagogically inappropriate, and prompted the AI to regenerate revised versions addressing these deficiencies. Similarly, Lee and Shin [33] adopted a structured expert-in-the-loop approach in which prompts were iteratively refined through expert consultation, followed by separate expert evaluations of educational applicability and clinical representativeness, with each round informing subsequent prompt revisions.

#### Toward Standardized Evaluation Frameworks

With the rapid expansion of GenAI in health care, several reporting and methodological guidelines have been developed to improve transparency and reproducibility, eg, CONSORT-AI (Consolidated Standards of Reporting Trials-AI) [109] and SPIRIT-AI (Standard Protocol Items: Recommendations for Interventional Trials-AI) [110] for AI-based clinical trials; GAMER (Generative AI tools in Medical Research) [111] for GenAI use in medical research; STAGER (Standardized Testing and Assessment Guidelines for Evaluating Generative AI Reliability) [112] for benchmarking GenAI systems; or METRICS (Model, Evaluation, Timing, Range, Randomization, Individual factors, Count, and Specificity of prompts and language) [113] for GenAI studies in health care education. These frameworks primarily aimed to standardize study reporting and methodological description, rather than provide criteria for evaluating AI-generated educational content itself.

More recently, Olla et al [114] proposed the AI-Integrated Method for Simulation (AIMS), a dual-phase framework for evaluating AI chatbot simulations in nursing education. Unlike reporting guidelines, AIMS evaluates both prompt design (clinical and content credibility, educational alignment, persona clarity, inclusivity, transparency, and personalization) and chatbot performance (credibility and accuracy, user experience, feedback quality, user agency, equity and accessibility, transparency, learning outcomes, and interactivity). Although developed specifically for AI-powered simulation chatbots, this framework illustrates the growing recognition that structured, multidimensional evaluation is needed for AI-generated educational materials.

Consistent with this emerging literature, our review found that evaluations of LLM-generated case-based educational materials relied on heterogeneous, locally defined criteria, most often based on small expert panels, limiting comparability across studies and cumulative evidence building. Based on the recurring evaluation domains identified in our review, we propose that health professions educators and researchers use a minimum evaluation set for LLM-generated case-based educational materials that includes (1) clinical accuracy and alignment with current guidelines, (2) clinical realism, (3) educational alignment (with learning objectives and student level), (4) structural quality (adherence to construction rules, clarity, coherence, and language quality), (5) pedagogical quality (appropriate difficulty, length, and student engagement), and (6) safety considerations, including the identification of biased or potentially harmful content. For assessment applications, this minimum set should be complemented by psychometric analyses, as well as student performance analysis and perceptions.

#### From Feasibility Studies to Responsible Curriculum Integration

As shown in our review, most studies on LLM-generated case-based materials in HPE were limited to feasibility or proof-of-concept, focusing on output plausibility or short-term evaluations rather than sustained curriculum integration. This aligns with broader evidence summarized by Basil et al [3], who identified persistent barriers to GenAI implementation in HPE, such as infrastructure and access constraints, cost, training gaps, ethical and legal concerns, and inconsistent output quality, factors that collectively slow down curriculum-level adoption. Addressing these challenges, Astbury et al [2] proposed a Generative AI in Health Professions Education (AI-HPE) checklist to guide responsible implementation, emphasizing institutional readiness, faculty and student preparedness, ethical governance, and ongoing quality assurance. At a more operational level, Kim et al [115] further argued that sustainable integration of AI-generated assessment items requires systematic preparation of faculty and examination bodies, including structured training pathways to develop AI and prompt literacy, clearly defined institutional workflows, shared prompt libraries aligned with local standards, and checklist-driven validation and calibration processes. These converging perspectives highlight that responsible curriculum integration requires not only evidence of validity and feasibility but also institutional readiness, faculty development, ongoing quality assurance, and structured human-AI workflows that maintain educator oversight.

### Limitations

This review has limitations related to its scope and methods. First, although a comprehensive search strategy was conducted across 7 major databases, some studies may have been missed due to language limitations (ie, English) and exclusion of gray literature and other databases (eg, Cochrane Library). Moreover, given the rapid development of LLMs in education, recent studies may not have been included; therefore, these findings should be interpreted as a picture of the current evidence. Second, consistent with the exploratory approach of scoping reviews, the methodological quality (including study bias) of the studies was not formally assessed, which could limit conclusions regarding the strength of the evidence. Additionally, the included studies [25-96] were heterogeneous regarding their design, educational settings, generation approaches, and outcome measures, limiting direct comparisons and conclusions about which approaches may improve the quality, reliability, or educational usefulness of LLM-generated materials. Future systematic reviews and meta-analyses could investigate the comparative effectiveness of different generation and refinement approaches. Finally, the review focused only on text-based case materials to maintain clarity and comparability. Studies involving multimodal case generation, such as images or videos, were outside the scope of this review.

### Conclusions

This scoping review provides a comprehensive synthesis of the rapidly evolving literature on the use of LLMs to generate case-based materials in HPE. Unlike previous reviews that examined GenAI across multiple educational apps or focused primarily on clinical cases, this review integrates evidence across a broad range of case-based materials while also synthesizing generation approaches, prompting strategies, evaluation methods, reported findings, and challenges. The mapped evidence suggests that LLMs have the potential to support the development of case-based materials, but current approaches continue to require expert oversight because of recurring concerns regarding clinical accuracy, educational quality, psychometric performance, clinical realism, and bias. The review also identifies important evidence gaps, including the predominance of studies in undergraduate medical education, limited evidence across other health professions, the reliance on small expert-based evaluations, limited adoption of advanced generation approaches, and the absence of standardized evaluation frameworks. By synthesizing the current evidence and identifying methodological and evidence gaps, this review provides educators, curriculum developers, and researchers with a structured overview of this rapidly evolving field and highlights priorities for future research. As evidence and LLMs continue to evolve, the findings of this review may inform the development of more robust generation workflows, more consistent evaluation approaches, and the responsible use of LLM-generated case-based materials in HPE.

## Supplementary material

10.2196/94795 Multimedia Appendix 1 Search Strategy.

10.2196/94795 Multimedia Appendix 2 Data charting table of included studies.

10.2196/94795 Checklist 1 PRISMA-ScR (Preferred Reporting Items for Systematic Reviews and Meta-Analyses extension for Scoping Reviews) checklist.

10.2196/94795 Checklist 2 PRISMA-S (Preferred Reporting Items for Systematic Reviews and Meta-Analyses Literature Search Extension) checklist.

## Abbreviations

AIMS: AI-Integrated Method for Simulation
CONSORT-AI: Consolidated Standards of Reporting Trials-AI
CoT: chain-of-thought
GAMER: Generative AI tools in Medical Research
GenAI: Generative AI
GPT: Generative Pretrained Transformer
HPE: Health Professions Education
LLM: large language model
LQBTQIA+: Lesbian, Gay, Bisexual, Transgender, Queer or Questioning, Intersex, and Asexual
MCQ: multiple-choice question
METRICS: Model, Evaluation, Timing, Range, Randomization, Individual factors, Count, and Specificity of prompts and language
NBME: National Board of Medical Examiners
OSCE: Objective Structured Clinical Examination
OSPE: Objective Structured Practical Examination
PBL: problem-based learning
PRISMA-S: Preferred Reporting Items for Systematic Reviews and Meta-Analyses Literature Search Extension
PRISMA-ScR: Preferred Reporting Items for Systematic Reviews and Meta-Analyses extension for Scoping Reviews
RAG: retrieval-augmented generation
SCT: Script Concordance Test
SPIRIT-AI: Standard Protocol Items: Recommendations for Interventional Trials-AI
STAGER: Standardized Testing and Assessment Guidelines for Evaluating Generative AI Reliability
