# Supplementary material for: Generation of Case-Based Materials Using Large Language Models in Health Professions Education: Scoping Review
Source: JMIR Med Educ. 2026 Nov 3;12:e94795. doi: 10.2196/94795 (PMC13730408; doi:10.2196/94795)
Supplement: Supplementary file 1 [file mededu-v12-e94795-s001.docx]

# **Search Strategy**

**Search period**

From January 1, 2017 to May 10, 2026.

**Search concepts**

The search strategy combined terms related to:

1. Generative artificial intelligence and large language models
2. Case-based educational materials
3. Health professions education

Controlled vocabulary terms (e.g., MeSH, Emtree) and free-text keywords were adapted for each database.

**Database:** PubMed (MEDLINE)
**Platform:** National Library of Medicine
**Date searched:** May 10, 2026
**Language restrictions:** None

| **Search #** | **Concept** | **Search query** |
| --- | --- | --- |
| #1 | Generative artificial intelligence/large language models | "large language model*"[tiab] OR "LLM"[tiab] OR "generative AI"[tiab] OR "generative artificial intelligence"[tiab] OR "genAI"[tiab] OR "generative pre-trained transformer"[tiab] OR "foundation model*"[tiab] OR "generative model*"[tiab]  OR  "ChatGPT"[tiab] OR "Chatbot*"[tiab] OR "GPT-*"[tiab] OR "Bard"[tiab] OR "Gemini"[tiab] OR "Claude"[tiab] OR "Mistral"[tiab] OR "Llama"[tiab] OR "PaLM"[tiab] OR "Ernie"[tiab] OR "DeepSeek"[tiab] OR "Sora"[tiab] OR "Copilot"[tiab] OR  "Artificial Intelligence"[Mesh] |
| #2 | Case-based materials | "clinical scenario*"[tiab] OR "simulation scenario*"[tiab] OR "case scenario"[tiab] OR "clinical vignette*"[tiab] OR "vignette*"[tiab] OR "clinical case*"[tiab] OR "case vignette*"[tiab] OR "clinical simulation*"[tiab]  OR  "simulation-based"[tiab] OR "scenario-based"[tiab] OR "case generat*"[tiab] OR "scenario generat*"[tiab] OR "case-based"[tiab] OR "case-based learning"[tiab] OR "CBL"[tiab] OR "problem-based"[tiab] OR "problem-based learning"[tiab] OR "PBL"[tiab] OR "objective structured clinical examination*"[tiab] OR "OSCE*"[tiab] OR "Problem-Based Learning"[Mesh] |
| #3 | Health professions education | "medical education"[tiab] OR "health professions education"[tiab] OR "healthcare education"[tiab] OR "clinical education"[tiab] OR "medical training"[tiab] OR "health professions training"[tiab]  OR  "medical student*"[tiab] OR "nursing student*"[tiab] OR "dental student*"[tiab] OR "pharmacy student*"[tiab] OR "physician assistant student*"[tiab] OR "PA student*"[tiab] OR "physician associate student*"[tiab] OR "physiotherap* student*"[tiab] OR "physical therap* student*"[tiab] OR "occupational therap* student*"[tiab] OR (speech[tiab] AND language[tiab] AND therap*[tiab] AND (student*[tiab] OR educat*[tiab])) OR "dietetic* student*"[tiab] OR "nutrition student*"[tiab] OR "paramedic* student*"[tiab] OR "paramedicine student*"[tiab] OR "midwif* student*"[tiab] OR "allied health student*"[tiab] OR "health profession* student*"[tiab]  OR  "nursing education"[tiab] OR "dental education"[tiab] OR "pharmacy education"[tiab] OR "physician assistant education"[tiab] OR "physician associate education"[tiab] OR "physiotherap* education"[tiab] OR "physical therap* education"[tiab] OR "occupational therap* education"[tiab] OR "dietetic* education"[tiab] OR "nutrition education"[tiab] OR "paramedic* education"[tiab] OR "paramedicine education"[tiab] OR "midwif* education"[tiab] OR "allied health education"[tiab] OR "health profession* education"[tiab] OR "Education, Professional"[Mesh] OR "Students, Health Occupations"[Mesh] |
| #4 | Combined search | #1 AND #2 AND #3 |
| #5 | Date limit | #4 AND ("2017/01/01"[Date - Publication] : "2026/05/10"[Date - Publication]) |

*tiab = title/abstract field*

*Mesh = Medical Subject Headings*

**Database:** Embase
**Platform:** Elsevier
**Date searched:** May 10, 2026

**Language restrictions:** None

| **Search #** | **Concept** | **Search query** |
| --- | --- | --- |
| #1 | Generative artificial intelligence/large language models | 'large language model*':ti,ab OR 'llm':ti,ab OR 'generative ai':ti,ab OR 'generative artificial intelligence':ti,ab OR 'genai':ti,ab OR 'generative pre-trained transformer':ti,ab OR 'foundation model*':ti,ab OR 'generative model*':ti,ab  OR  'chatgpt':ti,ab OR 'chatbot*':ti,ab OR 'gpt-3':ti,ab OR 'gpt-3.5':ti,ab OR 'gpt-4':ti,ab OR 'gpt-4.5':ti,ab OR 'gpt-5*':ti,ab OR 'bard':ti,ab OR 'gemini':ti,ab OR 'claude':ti,ab OR 'mistral':ti,ab OR 'llama':ti,ab OR 'palm':ti,ab OR 'ernie':ti,ab OR 'deepseek':ti,ab OR 'sora':ti,ab OR 'copilot':ti,ab OR 'artificial intelligence'/exp |
| #2 | Case-based materials | 'clinical scenario*':ti,ab OR 'simulation scenario*':ti,ab OR 'case scenario':ti,ab OR 'clinical vignette*':ti,ab OR 'vignette*':ti,ab OR 'clinical case*':ti,ab OR 'case vignette*':ti,ab OR 'clinical simulation*':ti,ab  OR  'simulation-based':ti,ab OR 'scenario-based':ti,ab OR 'case generat*':ti,ab OR 'scenario generat*':ti,ab OR 'case-based':ti,ab OR 'case-based learning':ti,ab OR 'cbl':ti,ab OR 'problem-based':ti,ab OR 'problem-based learning':ti,ab OR 'pbl':ti,ab OR 'objective structured clinical examination*':ti,ab OR 'osce*':ti,ab OR 'problem based learning'/exp OR 'case based learning'/exp |
| #3 | Health professions education | 'medical education':ti,ab OR 'health professions education':ti,ab OR 'healthcare education':ti,ab OR 'clinical education':ti,ab OR 'medical training':ti,ab OR 'health professions training':ti,ab  OR  'medical student*':ti,ab OR 'nursing student*':ti,ab OR 'dental student*':ti,ab OR 'pharmacy student*':ti,ab OR 'physician assistant student*':ti,ab OR 'pa student*':ti,ab OR 'physician associate student*':ti,ab OR 'physiotherap* student*':ti,ab OR 'physical therap* student*':ti,ab OR 'occupational therap* student*':ti,ab OR (speech:ti,ab AND language:ti,ab AND therap*:ti,ab AND (student*:ti,ab OR educat*:ti,ab)) OR 'dietetic* student*':ti,ab OR 'nutrition student*':ti,ab OR 'paramedic* student*':ti,ab OR 'paramedicine student*':ti,ab OR 'midwif* student*':ti,ab OR 'allied health student*':ti,ab OR 'health profession* student*':ti,ab  OR  'nursing education':ti,ab OR 'dental education':ti,ab OR 'pharmacy education':ti,ab OR 'physician assistant education':ti,ab OR 'physician associate education':ti,ab OR 'physiotherap* education':ti,ab OR 'physical therap* education':ti,ab OR 'occupational therap* education':ti,ab OR 'dietetic* education':ti,ab OR 'nutrition education':ti,ab OR 'paramedic* education':ti,ab OR 'paramedicine education':ti,ab OR 'midwif* education':ti,ab OR 'allied health education':ti,ab OR 'health profession* education':ti,ab OR 'vocational education'/exp OR 'health student'/exp OR 'medical profession'/exp |
| #4 | Combined search | #1 AND #2 AND #3 |
| #5 | Date limit | #4 AND [2017-2026]/py |

*ti = title field*

*ab = abstract field*

*exp = exploded Emtree subject heading*

**Database:** Scopus
**Platform:** Elsevier
**Date searched:** May 10, 2026
**Language restrictions:** None

| **Search #** | **Concept** | **Search query** |
| --- | --- | --- |
| #1 | Generative artificial intelligence/large language models | TITLE-ABS-KEY("large language model*" OR "LLM" OR "generative AI" OR "generative artificial intelligence" OR "genAI" OR "generative pre-trained transformer" OR "foundation model*" OR "generative model*"  OR  "ChatGPT" OR "Chatbot*" OR "GPT-*" OR "Bard" OR "Gemini" OR "Claude" OR "Mistral" OR "Llama" OR "PaLM" OR "Ernie" OR "DeepSeek" OR "Sora" OR "Copilot" OR "artificial intelligence") |
| #2 | Case-based materials | TITLE-ABS-KEY("clinical scenario*" OR "simulation scenario*" OR "case scenario" OR "clinical vignette*" OR "vignette*" OR "clinical case*" OR "case vignette*" OR "clinical simulation*"  OR  "simulation-based" OR "scenario-based" OR "case generat*" OR "scenario generat*" OR "case-based" OR "case-based learning" OR "CBL" OR "problem-based" OR "problem-based learning" OR "PBL" OR "objective structured clinical examination*" OR "OSCE*") |
| #3 | Health professions education | TITLE-ABS-KEY("medical education" OR "health professions education" OR "healthcare education" OR "clinical education" OR "medical training" OR "health professions training"  OR  "medical student*" OR "nursing student*" OR "dental student*" OR "pharmacy student*" OR "physician assistant student*" OR "PA student*" OR "physician associate student*" OR "physiotherap* student*" OR "physical therap* student*" OR "occupational therap* student*" OR (speech AND language AND therap* AND (student* OR educat*)) OR "dietetic* student*" OR "nutrition student*" OR "paramedic* student*" OR "paramedicine student*" OR "midwif* student*" OR "allied health student*" OR "health profession* student*"  OR  "nursing education" OR "dental education" OR "pharmacy education" OR "physician assistant education" OR "physician associate education" OR "physiotherap* education" OR "physical therap* education" OR "occupational therap* education" OR "dietetic* education" OR "nutrition education" OR "paramedic* education" OR "paramedicine education" OR "midwif* education" OR "allied health education" OR "health profession* education") |
| #4 | Combined search | #1 AND #2 AND #3 |
| #5 | Date limit | PUBYEAR AFT 2016 |

*TITLE-ABS-KEY = title, abstract, and author keywords fields*

**Database:** Web of Science Core Collection
**Platform:** Clarivate
**Date searched:** May 10, 2026
**Language restrictions:** None

| **Search #** | **Concept** | **Search query** |
| --- | --- | --- |
| #1 | Generative artificial intelligence/large language models | TS=("large language model*" OR "LLM" OR "generative AI" OR "generative artificial intelligence" OR "genAI" OR "generative pre-trained transformer" OR "foundation model*" OR "generative model*"  OR  "ChatGPT" OR "Chatbot*" OR "GPT-*" OR "Bard" OR "Gemini" OR "Claude" OR "Mistral" OR "Llama" OR "PaLM" OR "Ernie" OR "DeepSeek" OR "Sora" OR "Copilot" OR "artificial intelligence") |
| #2 | Case-based materials | TS=("clinical scenario*" OR "simulation scenario*" OR "case scenario" OR "clinical vignette*" OR "vignette*" OR "clinical case*" OR "case vignette*" OR "clinical simulation*"  OR  "simulation-based" OR "scenario-based" OR "case generat*" OR "scenario generat*" OR "case-based" OR "case-based learning" OR "CBL" OR "problem-based" OR "problem-based learning" OR "PBL" OR "objective structured clinical examination*" OR "OSCE*") |
| #3 | Health professions education | TS=("medical education" OR "health professions education" OR "healthcare education" OR "clinical education" OR "medical training" OR "health professions training"  OR  "medical student*" OR "nursing student*" OR "dental student*" OR "pharmacy student*" OR "physician assistant student*" OR "PA student*" OR "physician associate student*" OR "physiotherap* student*" OR "physical therap* student*" OR "occupational therap* student*" OR (speech AND language AND therap* AND (student* OR educat*)) OR "dietetic* student*" OR "nutrition student*" OR "paramedic* student*" OR "paramedicine student*" OR "midwif* student*" OR "allied health student*" OR "health profession* student*"  OR  "nursing education" OR "dental education" OR "pharmacy education" OR "physician assistant education" OR "physician associate education" OR "physiotherap* education" OR "physical therap* education" OR "occupational therap* education" OR "dietetic* education" OR "nutrition education" OR "paramedic* education" OR "paramedicine education" OR "midwif* education" OR "allied health education" OR "health profession* education") |
| #4 | Combined search | #1 AND #2 AND #3 |
| #5 | Date limit | Publication years: 2026 or 2025 or 2024 or 2023 or 2022 or 2021 or 2020 or 2019 or 2018 or 2017 |

*TS = Topic search (title, abstract, author keywords, and Keywords Plus)*

**Database:** CINAHL Ultimate
**Platform:** EBSCOhost
**Date searched:** May 10, 2026
**Language restrictions:** None

| **Search #** | **Concept** | **Search query** |
| --- | --- | --- |
| #1 | Generative artificial intelligence/large language models | XB((MH "Artificial Intelligence+") OR "large language model*" OR "LLM" OR "generative AI" OR "generative artificial intelligence" OR "genAI" OR "generative pre-trained transformer" OR "foundation model*" OR "generative model*" OR  "ChatGPT" OR "Chatbot*" OR "GPT-*" OR "Bard" OR "Gemini" OR "Claude" OR "Mistral" OR "Llama" OR "PaLM" OR "Ernie" OR "DeepSeek" OR "Sora" OR "Copilot") |
| #2 | Case-based materials | XB((MH "Problem-Based Learning+") OR "clinical scenario*" OR "simulation scenario*" OR "case scenario" OR "clinical vignette*" OR "vignette*" OR "clinical case*" OR "case vignette*" OR "clinical simulation*"  OR  "simulation-based" OR "scenario-based" OR "case generat*" OR "scenario generat*" OR "case-based" OR "case-based learning" OR "CBL" OR "problem-based" OR "problem-based learning" OR "PBL" OR "objective structured clinical examination*" OR XB "OSCE*") |
| #3 | Health professions education | XB((MH "Education, Professional+") OR (MH "Students, Health Occupations+") OR "medical education" OR "health professions education" OR "healthcare education" OR "clinical education" OR "medical training" OR "health professions training"  OR  "medical student*" OR "nursing student*" OR "dental student*" OR "pharmacy student*" OR "physician assistant student*" OR "PA student*" OR "physician associate student*" OR "physiotherap* student*" OR "physical therap* student*" OR "occupational therap* student*" OR (speech AND language AND therap* AND (student* OR educat*)) OR "dietetic* student*" OR "nutrition student*" OR "paramedic* student*" OR "paramedicine student*" OR "midwif* student*" OR "allied health student*" OR "health profession* student*"  OR  "nursing education" OR "dental education" OR "pharmacy education" OR "physician assistant education" OR "physician associate education" OR "physiotherap* education" OR "physical therap* education" OR "occupational therap* education" OR "dietetic* education" OR "nutrition education" OR "paramedic* education" OR "paramedicine education" OR "midwif* education" OR "allied health education" OR "health profession* education") |
| #4 | Combined search | #1 AND #2 AND #3 |
| #5 | Date limit | Publication Date: 01/2017 – 05/2026 |

*MH = CINAHL Headings (exploded subject heading)
XB = title or abstract fields*

**Database:** IEEE Xplore
**Platform:** IEEE
**Date searched:** May 10, 2026
**Language restrictions:** None

| **Search #** | **Concept** | **Search query** |
| --- | --- | --- |
| #1 | Generative artificial intelligence/large language models | ("large language model*" OR "LLM" OR "generative AI" OR "generative artificial intelligence" OR "genAI" OR "generative pre-trained transformer" OR "foundation model" OR "generative model"  OR  "ChatGPT" OR "chatbot*" OR "GPT-3" OR "GPT-3.5" OR "GPT-4" OR "GPT-4.5" OR "GPT-5" OR "Bard" OR "Gemini" OR "Claude" OR "Mistral" OR "Llama" OR "PaLM" OR "Ernie" OR "DeepSeek" OR "Sora" OR "Copilot" OR "artificial intelligence") |
| #2 | Case-based materials | ("clinical scenario*" OR "simulation scenarios" OR "case scenario" OR "clinical vignette*" OR "vignette" OR "clinical case*" OR "case vignette" OR "clinical simulation"  OR  "simulation-based" OR "scenario-based" OR "case generat*" OR "scenario generation" OR "case-based" OR "case-based learning" OR "CBL" OR "problem-based" OR "problem-based learning" OR "PBL" OR "objective structured clinical examination" OR "OSCE") |
| #3 | Health professions education | ("medical education" OR "health professions education" OR "healthcare education" OR "clinical education" OR "medical training" OR "health professions training"  OR  "medical student*" OR "nursing students" OR "dental students" OR "pharmacy students" OR "physician assistant students" OR "PA students" OR "physician associate students" OR "physical therap* students" OR "occupational therapy students" OR ("speech" AND "language" AND therap* AND (students OR education)) OR "dietetic students" OR "nutrition students" OR "paramedic students" OR "paramedicine students" OR "midwifery students" OR "allied health students" OR "health profession* students"  OR  "nursing education" OR "dental education" OR "pharmacy education" OR "physician assistant education" OR "physician associate education" OR "physical therapy education" OR "occupational therapy education" OR "dietetic education" OR "nutrition education" OR "paramedic education" OR "paramedicine education" OR "midwifery education" OR "allied health education" OR "health professions education") |
| #4 | Combined search | #1 AND #2 AND #3 |
| #5 | Date limit | Range : 2017– 2026 |

**Database:** ERIC
**Platform:** ProQuest
**Date searched:** May 10, 2026
**Language restrictions:** None

| **Search #** | **Concept** | **Search query** |
| --- | --- | --- |
| #1 | Generative artificial intelligence/large language models | MAINSUBJECT.EXACT.EXPLODE("Artificial Intelligence") OR NOFT("large language model*") OR NOFT("LLM") OR NOFT("generative AI") OR NOFT("generative artificial intelligence") OR NOFT("genAI") OR NOFT("generative pre-trained transformer") OR NOFT("foundation model*") OR NOFT("generative model*")  OR  NOFT("ChatGPT") OR NOFT("Chatbot*") OR NOFT("GPT-*") OR NOFT("Bard") OR NOFT("Gemini") OR NOFT("Claude") OR NOFT("Mistral") OR NOFT("Llama") OR NOFT("PaLM") OR NOFT("Ernie") OR NOFT("DeepSeek") OR NOFT("Sora") OR NOFT("Copilot") |
| #2 | Case-based materials | MAINSUBJECT.EXACT.EXPLODE("Problem Based Learning") OR MAINSUBJECT.EXACT.EXPLODE("Case Method (Teaching Technique)") OR MAINSUBJECT.EXACT.EXPLODE("Vignettes") OR NOFT("clinical scenario*") OR NOFT("simulation scenario*") OR NOFT("case scenario") OR NOFT("clinical vignette*") OR NOFT("vignette*") OR NOFT("clinical case*") OR NOFT("case vignette*") OR NOFT("clinical simulation*")  OR  NOFT("simulation-based") OR NOFT("scenario-based") OR NOFT("case generat*") OR NOFT("scenario generat*") OR NOFT("case-based") OR NOFT("case-based learning") OR NOFT("CBL") OR NOFT("problem-based") OR NOFT("problem-based learning") OR NOFT("PBL") OR NOFT("objective structured clinical examination*") OR NOFT("OSCE*") |
| #3 | Health professions education | MAINSUBJECT.EXACT.EXPLODE("Medical Education") OR MAINSUBJECT.EXACT.EXPLODE("Allied Health Occupations Education") OR MAINSUBJECT.EXACT.EXPLODE("Nursing Education") OR MAINSUBJECT.EXACT.EXPLODE("Pharmaceutical Education") OR NOFT("medical education") OR NOFT("health professions education") OR NOFT("healthcare education") OR NOFT("clinical education") OR NOFT("medical training") OR NOFT("health professions training")  OR  NOFT("medical student*") OR NOFT("nursing student*") OR NOFT("dental student*") OR NOFT("pharmacy student*") OR NOFT("physician assistant student*") OR NOFT("PA student*") OR NOFT("physician associate student*") OR NOFT("physiotherap* student*") OR NOFT("physical therap* student*") OR NOFT("occupational therap* student*") OR (NOFT("speech") AND NOFT("language") AND NOFT("therap*") AND (NOFT("student*") OR NOFT("educat*"))) OR NOFT("dietetic* student*") OR NOFT("nutrition student*") OR NOFT("paramedic* student*") OR NOFT("paramedicine student*") OR NOFT("midwif* student*") OR NOFT("allied health student*") OR NOFT("health profession* student*")  OR  NOFT("nursing education") OR NOFT("dental education") OR NOFT("pharmacy education") OR NOFT("physician assistant education") OR NOFT("physician associate education") OR NOFT("physiotherap* education") OR NOFT("physical therap* education") OR NOFT("occupational therap* education") OR NOFT("dietetic* education") OR NOFT("nutrition education") OR NOFT("paramedic* education") OR NOFT("paramedicine education") OR NOFT("midwif* education") OR NOFT("allied health education") OR NOFT("health profession* education") |
| #4 | Combined search | #1 AND #2 AND #3 |
| #5 | Date limit | Publication date: 2017–2026 |

*MAINSUBJECT.EXACT.EXPLODE = searches an exact ERIC descriptor and all narrower related descriptors in the ERIC thesaurus hierarchy
NOFT = anywhere except full text*
